# MYEOV (*myeloma overexpressed gene*) drives colon cancer cell migration and is regulated by PGE_2_

**DOI:** 10.1186/1756-9966-29-81

**Published:** 2010-06-22

**Authors:** Garrett Lawlor, Peter P Doran, Padraic MacMathuna, David W Murray

**Affiliations:** 1 Gastrointestinal Unit, Mater Misericordiae University Hospital, Eccles Street, Dublin 7, Ireland; 2 UCD Clinical Research Centre, UCD School of Medicine & Medical Sciences, UCD, 21 Nelson St, Dublin 7, Ireland

## Abstract

**Introduction:**

We have previously reported that Myeov (MYEloma OVerexpressed gene) expression is enhanced in colorectal cancer (CRC) and that it promotes CRC cell proliferation and invasion. The role of Myeov in CRC migration is unclear. ProstaglandinE2 (PGE _2_) is a known factor in promoting CRC carcinogenesis. The role of PGE _2 _in modulating Myeov expression has also not been defined.

**Aim:**

To assess the role of Myeov expression in CRC cell migration and to evaluate the role of PGE _2 _in Myeov bioactivity.

**Methods:**

siRNA mediated Myeov knockdown was achieved in T84 CRC cells. Knockdown was assessed using quantitative real time PCR. The effect of knockdown on CRC cell migration was assessed using a scratch wound healing assay. Separately, T84 cells were treated with PGE _2 _(0.00025 μ M, 0.1 μ M and 1 μ M) from 30 min to 3 hours and the effect on Myeov gene expression was assessed using real time PCR.

**Results:**

Myeov knockdown resulted in a significant reduction in CRC cell migration, observable as early as 12 hours (P < 0.05) with a 39% reduction compared to control at 36 hours (p < 0.01). Myeov expression was enhanced after treatment with PGE _2_, with the greatest effect seen at 60 mins for all 3 PGE _2 _doses. This response was dose dependent with a 290%, 550% & 1,000% increase in Myeov expression for 0.00025 μ M, 0.1 μ M and 1 μ M PGE _2 _respectively.

**Conclusion:**

In addition to promoting CRC proliferation and invasion, our findings indicate that Myeov stimulates CRC cell migration, and its expression may be PGE _2 _dependant.

## Introduction

Colorectal cancer is a heterogeneous disease arising from a complex series of molecular changes [1]. In 1990, Fearon and Vogelstein described the molecular basis of colorectal cancer as a multi-step model of carcinogenesis [2]. The model describes the accumulation of genetic events, each conferring a selective growth advantage to an affected colon cell, including inactivation of tumour suppressor genes and activation of oncogenes.

Using a bioinformatics approach we have identified genes with enhanced expression in colorectal cancer tissue [3,4]. Myeov, (MYEloma OVerexpressed gene) was initially noted for its association with a subset of multiple myeloma cell lines [4,5] and it has also been implicated in oesophageal squamous cell carcinomas [6] and breast cancer [7]. Myeov is co-amplified with cyclin D1, a known oncogene [5]. We have previously shown Myeov to play a role in gastric cancer cell proliferation and invasion [3].

Our group has demonstrated a role for Myeov in the pathogenesis of colorectal cancer (CRC), noting a 20-fold increase in Myeov expression in CRC in comparison with normal colorectal tissue [3]. We have also confirmed that Myeov is upregulated in CRC *ex vivo *using tissue from normal colonic mucosa, adenomas, and carcinomas [3]. Our *In vitro *RNA interference/knockdown studies, in which Myeov expression was inhibited, revealed a role for Myeov in driving CRC cell proliferation and invasion. However, the role of Myeov expression in CRC cell migration has not been elucidated. We hypothesise, because of its established role in tumour cell invasion, that Myeov is also important for tumour cell migration. The mechanism underlying Myeov expression remains unclear.

In an effort to identify upstream effectors of Myeov expression, we assessed the effect of Prostaglandin E2 (PGE _2_) on Myeov. PGE _2 _is a well-established mediator in cancer progression, particularly in CRC. It has been shown to enhance intestinal adenoma growth in ApcMin mice models of CRC [8]. Driven by COX-2, PGE _2 _can enhance tumour growth by binding its receptors and activating signalling pathways which control cellular proliferation, migration, apoptosis, and angiogenesis, key features of malignancy [9]. We investigated the possibility that PGE _2 _may mediate the enhanced expression of Myeov in CRC. Consequently, the objectives of our study were two-fold; firstly, to assess the role of Myeov gene knockdown on CRC cell migration *in vitro*; secondly, to evaluate the effect of PGE _2 _on Myeov mRNA expression in CRC.

## Materials and methods

### Cell culture

The T84 cell line obtained from the European collection of cell cultures was used in this study as it is an established in vitro experimental model of colorectal carcinoma. The cell were cultured in Dulbecco’s modified Eagle’s medium-F12, with 1 U/ml penicillin, 1 lg/ml streptomycin, and 10% fetal bovine serum under standard conditions.

### siRNA knockdown

The functional role of Myeov was assessed using gene knockdown with small interfering RNA (siRNA) designed and synthesized for Myeov knockdown (Qiagen Inc., CA, USA). The siRNA had the following sequences: Myeov sense, 50-GGA UGU AAG UUA UCA ACU A-30; Myeov antisense, 50-UAG UUG AUA ACU UAC AUC C-30. A chemically synthesized non-silencing siRNA duplex with the following sequence; sense, 50-UUC UCC GAA CGU GUC ACG U-30; antisense, 50-ACG UGA CAC GUU CGG AGA A-30 that had no known homology with any mammalian gene was used to control for non-specific silencing events. Gene knockdown was achieved in T84 cells. Briefly, 4 × 10 ^4 ^cells were incubated under standard conditions overnight. 5 μg of each siRNA was then mixed with 30 μl of RNAifect (Qiagen) and was added drop wise. Cells were incubated for 48 h again under standard conditions before being assayed.

### RNA preparation and PCR

TRIzol (Sigma-Aldrich, Ireland) was used to extract RNA from cells. Reverse transcription was achieved using AMV reverse transcriptase (Invitrogen Ltd., UK). Real-time RT-PCR was performed using a Rotor Gene (Corbett Research, Australia). GAPDH, which was amplified in parallel with the genes of interest, served as a housekeeping gene. All measurements were performed in triplicate. The oligonucleotide primers and probes employed in this study were: MYEOV forward primer: CCT AAA TCC AGC CAC GTC AT, reverse primer; GAC ACA CCA CGG AGA CAA TG, GAPDH forward primer: GAA GGT GAA GGT CGG AGT TC, reverse primer GAA GAT GGT GAT GGG ATT TC.

### Cell migration 'Scratch Assay'

Following Myeov knockdown, a "scratch" was placed in a confluent T84 cell monolayer using a 10 μl micropipette tip [10]. Cell migration over this wound scratch was monitored by photographing at 1, 6, 12, 24 and 36 hours. Subsequent image analysis involved measuring scratch width at 5 random points. Average scratch width and standard deviation was calculated for each time point. Cells were photographed using a × 10 objective lens. Carnoy software (Biovolution) was used to measure the pixel width of the scratches.

### The effects of PGE _2 _on Myeov expression

T84 CRC cells were treated with increasing concentrations (0.00025 μM, 0.1 μM, 1 μM) of PGE _2 _(Sigma Aldrich, Ireland) for 30, 60 and 180 mins. The concentrations of PGE _2 _used reflect the optimal in-vitro concentration to induce cellular responses as noted in a number of studies [11-14]. RNA extraction and real time PCR were performed as described above.

### Statistics

All analyses were performed independently in triplicate. Students paired t-test was used to compare groups with a P value < 0.05 indicating statistical significance.

## Results

### The effect of Myeov gene knockdown on CRC cell migration

**Figure 1:**
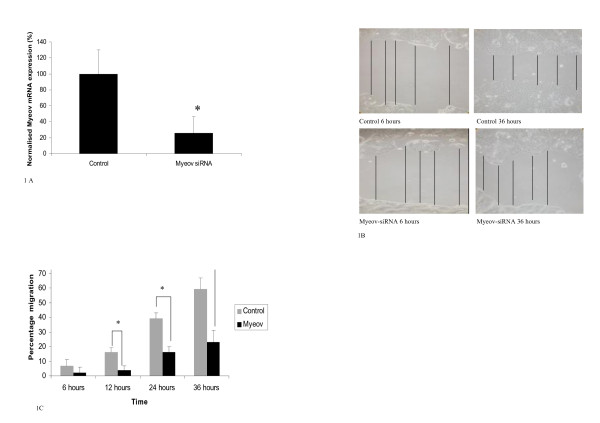
**(A) Confirmation of Myeov knockdown**. Myeov mRNA expression in control and siRNA treated cells was quantitated using real time PCR. (* = p < 0.05). (B) Representative images of the wound healing scratch assay. The lines represent measurements made to assess reduction in "scratch" width as a marker of migration. (C) Effect of Myeov knockdown on cell migration over time (* P < 0.05. ** P < 0.01).

In order to establish the role of Myeov in colorectal cancer cell migration we performed targeted knockdown using siRNA. A T84 cell line model of colorectal cancer was used. Successful knockdown of Myeov mRNA expression in T84 cells using siRNA was confirmed using quantitative real time PCR (Figure 1A). A 74% reduction in Myeov mRNA expression was observed in knockdown cells in comparison with control cells 48 hr post transfection (P < 0.05). In order to investigate the effect of Myeov depletion on T84 colorectal cancer cell migration, scratch wound healing assays were performed. Myeov knockdown resulted in decreased T84 colorectal cancer cell migration. Myeov knockdown resulted in a 25%, 41%, and 39% reduction in T84 colorectal cancer cell migration was observed at 12, 24 and 36 hrs respectively compared to control cells (P < 0.05) (Figure 1C).

### The effect of PGE_2 _on Myeov expression

**Figure 2:**
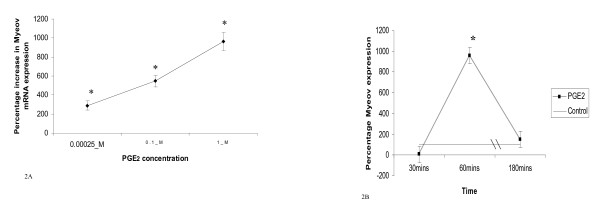
**The effect of PGE _2 _on Myeov expression**. (A) The % change in Myeov expression in T84 CRC cells treated with increasing doses of PGE _2 _at 60 mins in comparison with untreated cells (* = P < 0.05). (B) The time dependent effect of PGE _2 _on Myeov expression. T84 CRC cells were treated with 1 μM PGE _2 _and Myeov expression was assessed at various time points. Expression is represented as percentage change in expression in comparison with untreated control cells at each timepoint. (* = P < 0.05).

In order to investigate the effect of PGE _2 _on Myeov gene expression in colorectal cancer, T84 colorectal cancer cells were treated with varying doses of PGE _2 _for varying times in vitro and Myeov mRNA expression was monitored using quantitative real time PCR. Treatment of T84 cells with PGE _2 _for 24 hr resulted in increased Myeov expression however the maximum effect occurred at 60 mins (Figure 2A &2B). Furthermore this effect was dose-dependent. At 60 mins, 0.00025 μ M PGE _2 _increased Myeov gene expression by 289%, 0.1 μM PGE _2 _increased Myeov expression by 547% and 1.0 μM PGE _2 _increased Myeov expression by 961% (P < 0.05). Treatment with PGE _2 _for 30 min resulted in decreased Myeov expression with 1.0 μM treatment having a significant inhibitory effect, decreasing Myeov expression by 99% (P < 0.01) (Figure 2B).

## Discussion

Colorectal cancer has a significant morbidity and mortality, being the fourth most common cancer worldwide [15]. Defining the pathways that drive colorectal cancer will provide a better understanding of neoplastic progression, and may potentially identify targets for therapeutic intervention. Myeov expression has previously been shown to be enhanced in myeloma as well as breast, esophageal and gastric cancers [7,9]. We have employed Digital Differential Display (DDD) a bioinformatic tool, to identify Myeov as a novel colorectal cancer associated gene [3]. Briefly, we used DDD to compare expressed sequence tags (ESTs) between normal colorectal and cancer tissue, thereby identifying differentially expressed genes. Myeov was shortlisted for further investigation and we demonstrated enhanced Myeov expression in colorectal cancer and that it promotes tumour proliferation and invasion [3], key hallmarks of metastatic cancer. These datasets support the important role of Myeov in this disease.

Gene knockdown using siRNA represents an excellent tool to assess the functional importance of cancer related genes in vitro. We have previously employed siRNA to knockdown Myeov in colorectal and gastric cancer cell lines and have shown knockdown to result in decreased cell proliferation and invasion [3,9]. Using this technology, the current study further supports the functional importance of Myeov in CRC by showing that it drives colorectal cancer cell migration, a key process in the malignant phenotype. This data consolidates our previous reports that Myeov drives both proliferation and invasion. This new data illustrates a further role for Myeov in the motility of colorectal cancer cell and key hallmark of metastatic tumour cells.

Having established Myeov as a key player in CRC cell biology, we investigated whether Myeov was a downstream effector of COX/PGE _2 _bioactivity. PGE _2 _is a well established player in the progression of CRC and has been shown to induce increased proliferation, migration, and invasiveness of CRC cells [16]. We hypothesise that enhanced COX/PGE _2 _bioactivity in CRC leads to increased levels of Myeov and therefore increased invasion and migration. We have demonstrated in this study that treatment with PGE _2 _enhances the expression of Myeov. Although the signalling mechanisms connecting PGE _2 _signalling and Myeov transcription remain unknown, our findings support the hypothesis that Myeov is in part PGE _2 _regulated and contributes to the downstream oncogenic activity of COX. PGE _2 _has been shown to drive CRC cell migration and enhanced Myeov expression may at least in part mediate this process [16]. The precise signalling and transcriptional mechanisms at play here need to be further deciphered. It has been shown that PGE _2_-mediated CRC cell migration is related to the intracellular activation of EGFR by PGE _2 _[17]. Further work will clarify if Myeov expression is regulated by PGE _2 _in a similar manner. Interestingly, we also quantitated the levels of secreted PGE _2 _in Myeov knockdown and control cells however no significant difference was observed, confirming that the regulation of PGE _2 _expression is not downstream of Myeov bioactivity (data not shown).

These findings further define the role for Myeov bioactivity in colorectal carcinogenesis. Ongoing studies into Myeov expression will expand this pathway to reveal newer insights into colorectal cancer progression and possibly enable a potential therapeutic based on targeting Myeov.

## Competing interests

The authors declare that they have no competing interests.

## Authors' contributions

GL performed the experimental programme descried herein. He also prepared the manuscript. PMM acted as clinical liaison on this study and ensured the study was clinically relevant. He also read and proofed the finalised manuscript. PPD acted as a scientific liaison on this study and contributed to the experimental design. He also proofed the finalised manuscript. DWM conceived, designed and trouble-shooted the experimental programme described herein, he acted as a laboratory supervisor to GL and assisted in the preparation and proofing of this manuscript. All authors have read and approved the final manuscript.
